# Supplementary material for: Coulomb interactions between dipolar quantum fluctuations in van der Waals bound molecules and materials
Source: Nat Commun. 2021 Jan 8;12:137. doi: 10.1038/s41467-020-20473-w (PMC7794295; doi:10.1038/s41467-020-20473-w)
Supplement: Supplementary file 1 — Supplementary Information [file 41467_2020_20473_MOESM1_ESM.pdf]

Supplementary Material for

Coulomb Interactions between Dipolar Quantum Fluctuations in van der Waals Bound Molecules and Materials

Martin Stöhr[1], Mainak Sadhukhan[1,2], Yasmine S. Al-Hamdani[1,3], Jan Hermann[1], and Alexandre Tkatchenko*[1]

[1] *Department of Physics and Materials Science, University of Luxembourg, L-1511 Luxembourg*
[2] *Department of Chemistry, Indian Institute of Technology Kanpur, Kalyanpur, Kanpur-208 016, India*
[3] *Department of Chemistry, University of Zürich, CH-8057 Zürich, Switzerland*

Supplementary Methods

The damping function, f_{damp}^{AB}, for the beyond-dipolar potential entering the Dipole-Correlated Coulomb Singles (DCS) energy,

$$E_{\mathrm{DCS}} = \left\langle \Psi_{\mathrm{DC}} \middle| f_{\mathrm{damp}}^{AB} \left(V_{AB}^{\mathrm{Coul}} - V_{AB}^{\mathrm{dip}} \right) \middle| \Psi_{\mathrm{DC}} \right\rangle - \left\langle \Psi_0 \middle| f_{\mathrm{damp}}^{AB} \left(V_{AB}^{\mathrm{Coul}} - V_{AB}^{\mathrm{dip}} \right) \middle| \Psi_0 \right\rangle \ , \tag{1}$$

has been based on the Fermi-like range-separation in MBD:

$$f_{\mathrm{damp}}^{AB} = \left\{ 1 + \exp\left[-a \left(\frac{R_{AB}}{\beta \cdot \left(R_{\mathrm{vdW}}^{(A)} + R_{\mathrm{vdW}}^{(B)} \right)} - 1 \right) \right] \right\}^{-1} \ , \tag{2}$$

where $R_{\mathrm{vdW}}^{(A)}$ is the van der Waals (vdW) radius of atom A. To assure the same switching behavior and avoid skewing of the damping function, we have fixed the ratio between the two parameters, a and β, defining the damping function, which allows for a simple shift in the damping, see Figure 1.

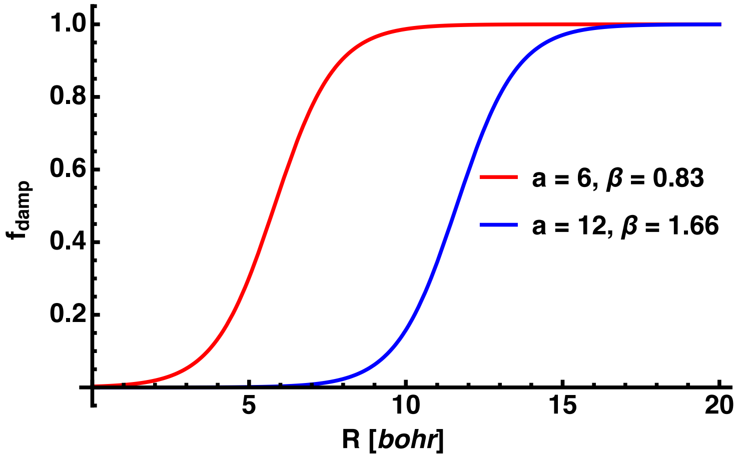

Figure 1: Shift of original MBD damping function (red) by keeping a fixed ratio of damping parameters.

*email: alexandre.tkatchenko@uni.lu

Supplementary Figures

Dipole-Correlated Coulomb Singles in small organic dimers. Small molecular dimers as contained in the S66 dataset, for example, represent a good approximation for two interacting bodies in isotropic vacuum. As such, contributions from DCS are expected to be negligible. Figure 2 shows E_{DCS} for the individual dimers contained in the S66 set in comparison to the full PBE+MBD+DCS interaction energy. As evident from the plot, DCS contributions, indeed, can be neglected for small organic dimers. Hence, the good performance of vdW models within the dipole limit, which is commonly reported for small organic dimers, is not affected by including DCS.

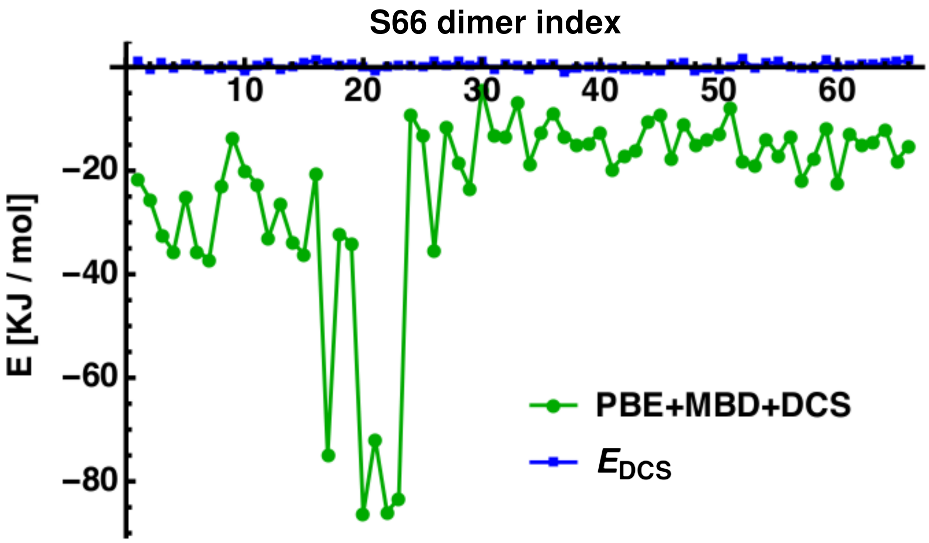

Figure 2: Dipole-Correlated Coulomb Singles binding energies (blue squares) for small molecular dimers as contained in the S66 dataset in comparison to full PBE+MBD+DCS interaction (green circles).

Qualitative descriptors for Dipole-Correlated Coulomb Singles. The DCS energy can be separated into charge polarization effects arising from long-range electron correlation and beyond-dipolar vdW interactions. Correspondingly, the displaced charge due to vdW dispersion as obtained within the MBD formalism resembles the relative trend in DCS interaction energies. Figure 3A shows a correlation plot of the displaced charge with the DCS contribution to the binding energy of a fullerene to various host molecules.

To further analyze geometric descriptors for spatial proximity, we tested a variety of power law summations of the atom-pairwise distances. As can be seen from Figure 3B, the sum of inverse distances to the power eight also provides a qualitative descriptor for E_{DCS}. Within the atom-pairwise formulation of vdW interactions, such a sum also represents dipole–quadrupole vdW contributions. This connection is merely coincidental, however. DCS reduce the binding energies in all cases, whereas extending MBD with atom-pairwise dipole–quadrupole vdW interaction ($-\mathrm{C}_8/\mathrm{R}^8$-potentials), would add additional, *attractive* interaction. The best correlation (*i.e.*, the least deviation from linear behavior) between E_{DCS} and power law summations can be found for the fifth power of inverse atom-pairwise distances (*cf.* Figure 3B). This can be rationalized by the fact that, as a leading-order term, dipolar electron correlation induces static quadrupole moments, whose interaction decays as R^{-5}. Further investigation of the general validity and origin of the observed correlations is subject to future studies.

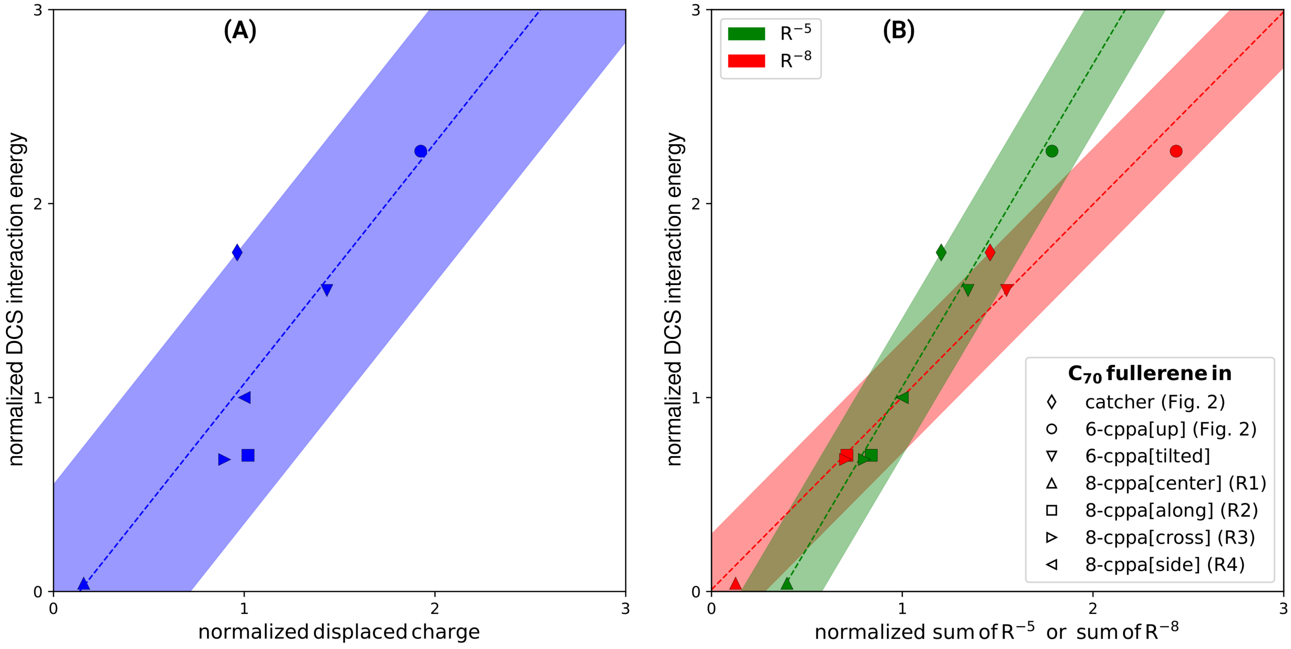

Figure 3: Correlation of displaced charge (A) and the sum of R^{-5}- or R^{-8}-terms (B) with interaction contributions from Dipole-Correlated Coulomb Singles for the C_{70}-fullerene in various host molecules.

Full van der Waals-inclusive DFT Energies for Xe_2 in CNTs. Figure 4 summarizes the full interaction energies for a Xe dimer inside (5,5)- and (6,6)-CNTs as obtained with PBE-DFT, PBE+MBD and PBE+MBD+DCS. The bare PBE interaction energy can thereby be considered to be negligible beyond *inter*-Xe distances of about 6 Å while it introduces the expected repulsive contribution at shorter separations. Overall, the qualitative nature of the intermediate- and long-range interaction reported in Figure 4 of the main manuscript is thus not altered by the addition of PBE-DFT energetics. Comparing the results for PBE+MBD and PBE+MBD with Dipole-Correlated Coulomb Singles, one can observe two important aspects. i) The well-defined meta-stable state for Xe_2 in the (5,5)-CNT observed for PBE+MBD (red line, middle plot) is counteracted by the Dipole-Correlated Coulomb Singles contribution, which ultimately leads to an cancellation of the local minimum (see bottom plot). Overall, the interaction energy inside the (5,5)-CNT is not characterized by a considerable meta-stable state upon inclusion of DCS. ii) Also in the case of the Xe dimer with increased polarizability ("1.5 α_{Xe}"), the notable minimum around 5.2 Å observed with PBE+MBD is almost completely attenuated once accounting for DCS contributions, while the complex oscillatory behavior between weak attraction and repulsion remains when including PBE-DFT energies.

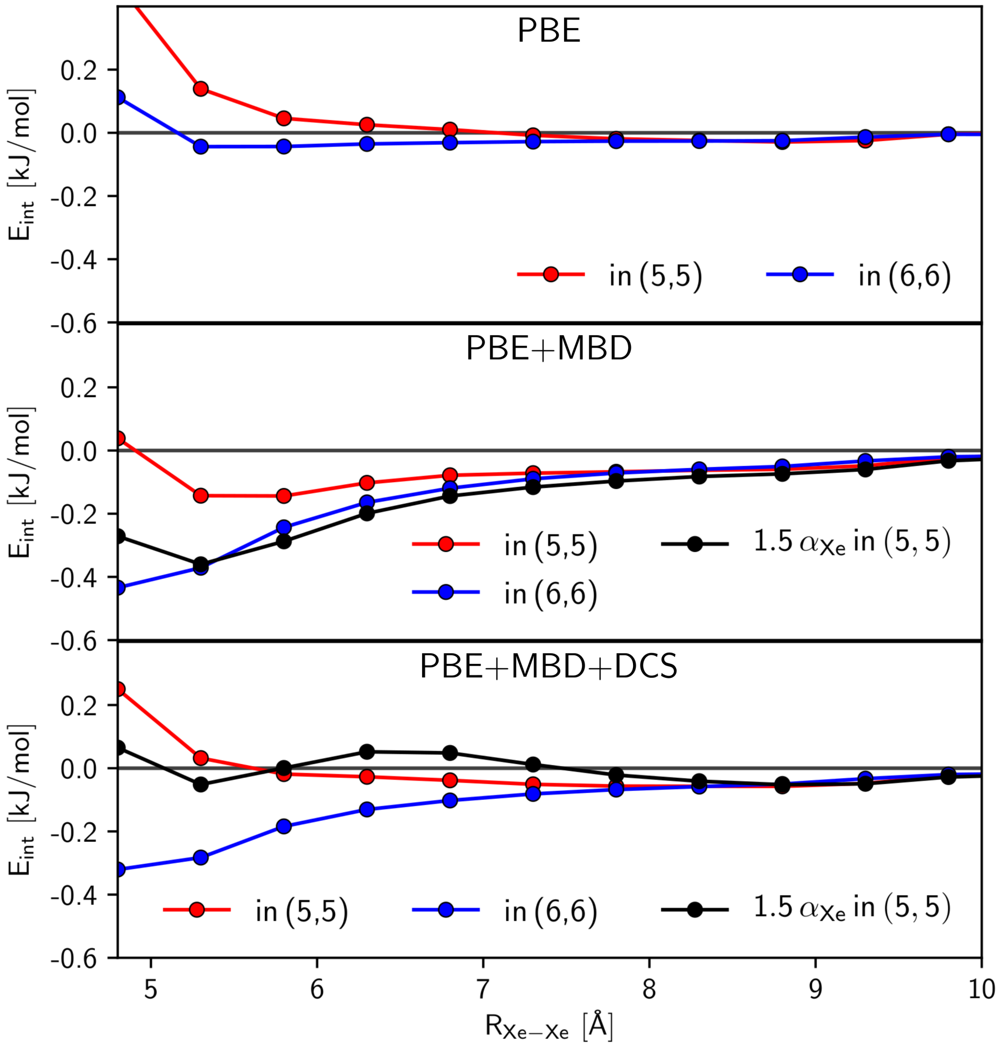

Figure 4: Full van der Waals-inclusive PBE energetics for Xe_2 inside (5,5)- and (6,6)-carbon nanotubes. *top:* Bare PBE interaction energy, *middle:* PBE+MBD energetics, *bottom:* PBE+MBD+DCS interaction.

Supplementary Tables

Table 1: **Many-Body Dispersion and Dipole-Correlated Coulomb Singles interaction energies of Xenon dimer in gas phase (gp) or confined within a (6,6)- or (5,5)-carbon nanotube.**

	$E_{\text{int}}^{(\text{MBD})}$ [kJ/mol]			$E_{\text{int}}^{(\text{DCS})}$ [kJ/mol]		
R [Å]	gp	in (6,6)	in (5,5)	gp	in (6,6)	in (5,5)
4.3	−1.5460	−0.8811	−0.6897	0.0894	0.1318	0.2424
4.8	−1.0603	−0.5464	−0.4344	0.0818	0.1135	0.2118
5.3	−0.6702	−0.3272	−0.2822	0.0647	0.0883	0.1750
5.8	−0.4149	−0.1998	−0.1896	0.0430	0.0590	0.1253
6.3	−0.2595	−0.1290	−0.1279	0.0234	0.0337	0.0753
6.8	−0.1660	−0.0881	−0.0885	0.0104	0.0172	0.0401
7.3	−0.1089	−0.0616	−0.0636	0.0040	0.0081	0.0207
7.8	−0.0733	−0.0449	−0.0483	0.0014	0.0037	0.0108
8.3	−0.0505	−0.0335	−0.0381	0.0005	0.0014	0.0053
8.8	−0.0356	−0.0255	−0.0304	0.0002	0.0004	0.0020
9.3	−0.0256	−0.0195	−0.0238	0.0001	0.0000	0.0002
9.8	−0.0187	−0.0151	−0.0180	0.0000	−0.0001	−0.0005
10.3	−0.0138	−0.0118	−0.0135	0.0000	0.0000	−0.0004
10.8	−0.0104	−0.0093	−0.0102	0.0000	0.0001	0.0000
11.3	−0.0079	−0.0075	−0.0079	0.0000	0.0001	0.0003
11.8	−0.0061	−0.0060	−0.0063	0.0000	0.0000	0.0003
